# Dietary or pharmacological inhibition of insulin-like growth factor-1 protects from renal ischemia-reperfusion injury in mice

**DOI:** 10.1016/j.isci.2024.111256

**Published:** 2024-10-28

**Authors:** Arnaud Lyon, Thomas Agius, Michael R. Macarthur, Kevin Kiesworo, Louis Stavart, Florent Allagnat, Sarah J. Mitchell, Leonardo V. Riella, Korkut Uygun, Heidi Yeh, Sebastien Déglise, Déla Golshayan, Alban Longchamp

**Affiliations:** 1 Department of Vascular Surgery, Lausanne University Hospital (CHUV), University of Lausanne (UNIL), Lausanne, Switzerland; 2 Transplantation Center and Transplantation Immunopathology Laboratory, Lausanne University Hospital (CHUV), University of Lausanne (UNIL), Lausanne, Switzerland; 3 Center for Engineering in Medicine, Department of Surgery, Massachusetts General Hospital, Harvard Medical School, Boston, MA, USA; 4 Transplant Center, Department of Surgery, Massachusetts General Hospital, Harvard Medical School, Boston, MA, USA; 5 Lewis-Sigler Institute for Integrative Genomics, Princeton University, Princeton, NJ, USA; 6 Ludwig Princeton Branch, Princeton University, Princeton, NJ, USA

**Keywords:** Pharmacology, Natural sciences, Biological sciences, Biochemistry, Physiology, Cellular physiology

## Abstract

One-week protein restriction (PR) limits ischemia-reperfusion (IR) damages and improves metabolic fitness. Similarly, longer-term calory restriction results in increased lifespan, partly via reduced insulin-like growth factor (IGF)-1. However, the influence of short-term PR on IGF-1 and its impact on IR are unknown. PR was achieved in mice via one-week carbohydrate loading and/or through a low-protein diet. PR decreased IGF-1 circulating levels as well as renal and hepatic expression. Upon renal IR, serum IGF-1 positively correlated with renal dysfunction and tissular damages, independently of sex and age. Exogenous IGF-1 administration abrogated PR benefits during IR, while IGF-1 receptor inhibition with linsitinib was protective. IGF-1 was associated with a reduction in forkhead box O (FoxO), and AMP-activated protein kinase (AMPK) signaling pathways previously demonstrated to improve IR resilience in various organs. These data support dietary or pharmacological reduction of IGF-1 signaling to mitigate IR injury prior to solid organ transplantation and beyond.

## Highlights

• IGF-1 levels positively correlate with severity following renal IR injury
• Pharmacological inhibition of IGF-1 signaling protects against renal IR injury
• Exogenous IGF-1 increases IR injury and abrogates protein restriction benefits
• IGF-1 downregulates FoxO and AMPK signaling pathways in the kidney

## Graphical abstract

**Figure:**
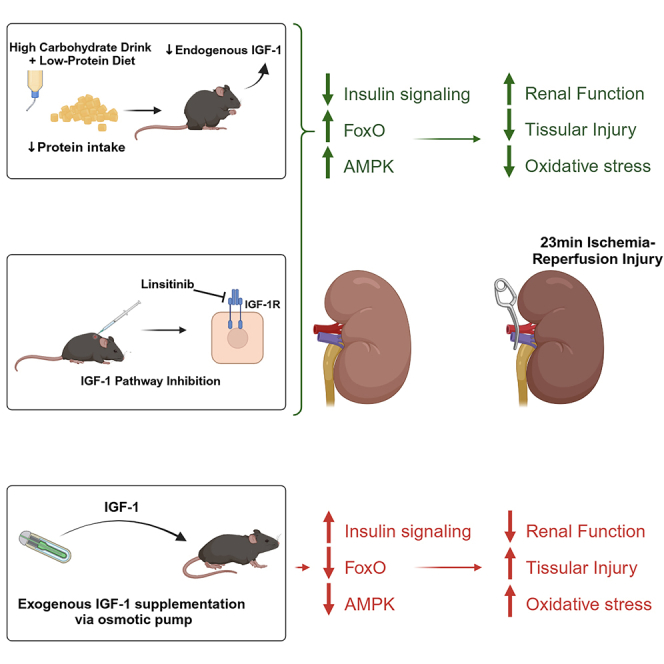

## Introduction

Ischemia-reperfusion (IR) injury is a pathological process involving the intermittent cessation of blood flow to an organ or tissue (ischemia) and subsequent restoration of blood flow (reperfusion).^1^^,^^2^ IR is inevitably associated with various medical/surgical interventions including solid organ transplantation, cardiovascular surgery, contrast-induced nephropathy, and pathological events such as myocardial infarction, stroke, or peripheral arterial disease.^1^^,^^2^ During ischemia, oxygen and nutrients supply to the cell is reduced, resulting in a reduction of ATP synthesis, accumulation of toxic by-products, intracellular electrolyte dysregulation and ultimately loss of membrane potential. Reperfusion exacerbates the damage through extensive reactive oxygen species (ROS) generation by reverse electron transport in the mitochondria^3^^,^^4^ ultimately leading to cell death and organ dysfunction.^5^ To date, there is no specific treatment to prevent or mitigate IR injury.

Protein restriction (PR), also known as protein dilution, is defined as reduced protein/amino acid intake without malnutrition or enforced energy restriction. PR is associated with a range of health benefits in animal models including improved glucose and lipid homeostasis.^6^^,^^7^^,^^8^^,^^9^^,^^10^ PR further increases resilience against acute stressors,^11^^,^^12^ and longevity.^13^^,^^14^^,^^15^^,^^16^ In most experimental models, PR is achieved by the isocaloric replacement of protein with carbohydrates in the food, increasing the dietary carbohydrate: protein ratio.^17^ Importantly, our team recently demonstrated that, in *ad libitum*-fed mice, replacement of water with a carbohydrate loading drink for one week drives reduction in solid food intake and voluntary dietary PR.^18^ In this context, carbohydrate loading induced PR resulted in protection from renal and hepatic IR injury.^18^ Similarly, in humans, low protein intake is associated with reduced all-cause mortality and improved cardiovascular risk factors profiles, including improved glucose and lipid homeostasis.^7^^,^^8^^,^^19^

Short-term (less than one week) PR acutely lowers serum insulin-like growth factor (IGF)-1 concentration in humans and in rodents.^20^ The benefits of reduced IGF-1 are thought to work through the activation of stress resistance pathways normally suppressed by insulin/IGF-1 signaling, for example, expression of antioxidant-encoding genes.^21^ Consistently, IGF-1 receptor (IGF-1R) inhibition reduced the levels of pro-inflammatory cytokines and chemokines associated with aging in mice.^22^^,^^23^ In contrast, the activation of the IGF-1R was shown to reduce cell death after IR injury in the kidney,^24^^,^^25^ heart^26^ and brain^27^ by activating the intracellular phosphoinositide 3-kinase/protein kinase B (PI3K/AKT) signaling pathway,^26^^,^^27^ by enhancing epidermal growth factor (EGFR) activation^25^ and by reducing protein catabolism and enhancing tissue repair.^24^

In this study, our aims were to: 1) Evaluate the association between PR, IGF-1, and renal IR injury. 2) Assess the benefits of pharmacological IGF-1R blockade before IR injury. 3) Identify the mechanisms and IGF-1 downstream targets during renal IR. We further hypothesized that PR reduces IGF-1 and that the pre-operative inhibition of the IGF-1 signaling pathway could replicate the benefits of PR and reduce IR injury.

## Results

### Insulin-like growth factor-1 is associated with protection from renal ischemia-reperfusion injury

**Figure 1:**
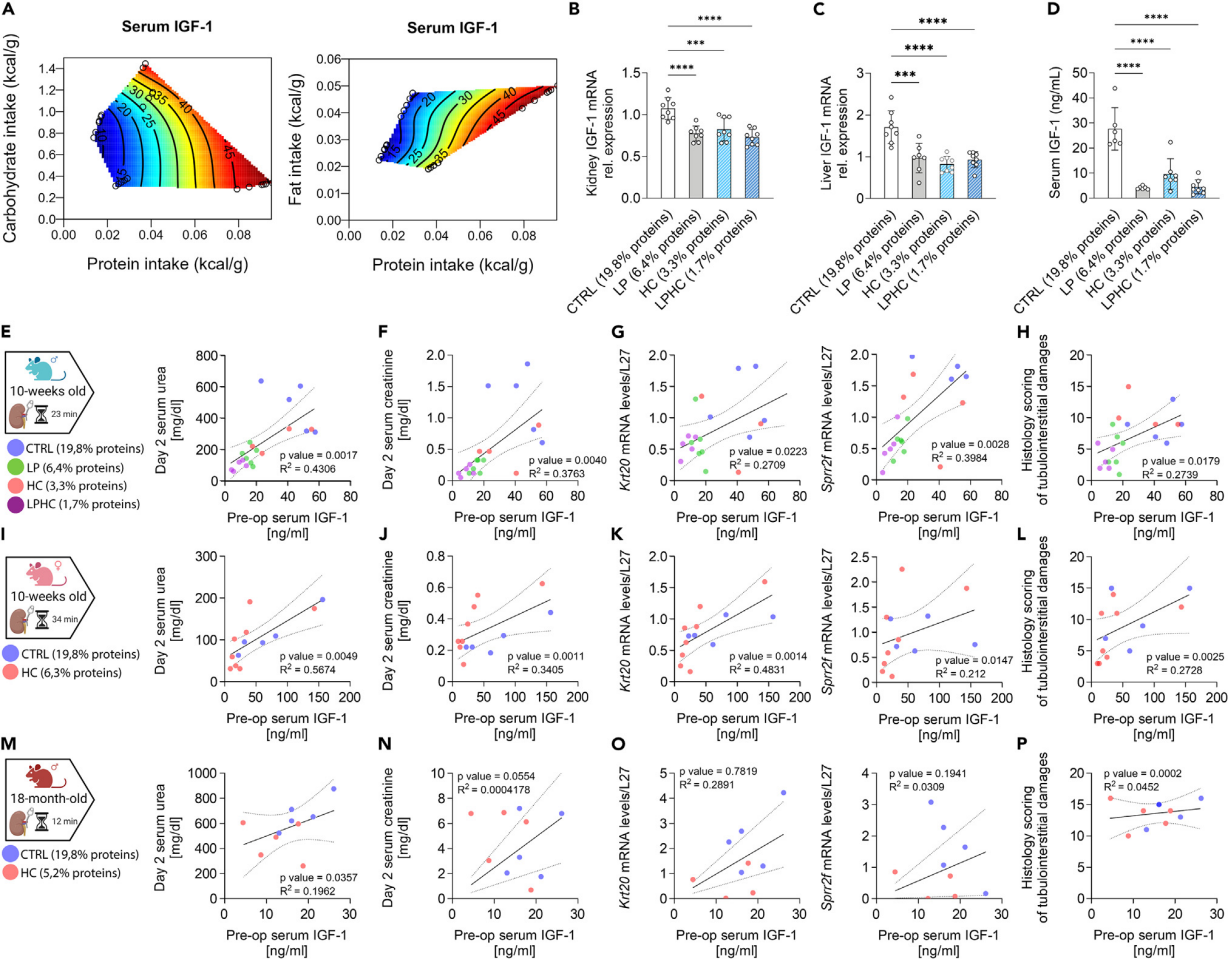
IGF1 correlates with sensitivity to ischemia-reperfusion injury during protein restriction (A) Response surface shows relationship between protein intake, carbohydrate intake (left), fat intake (right) and serum IGF-1 concentration in 10-week-old male mice. (B) Relative *Igf1* mRNA levels in kidneys and (C) in livers of 10-week-old male mice after 1 week preconditioning with the indicated diet. (D) Serum IGF-1 concentration for 10-week-old male mice after 1 week preconditioning with the indicated diet. (E) Experimental group (left) and correlation between preoperative serum IGF-1 concentration and urea levels (right), (F) serum creatinine (G), *Krt20* and *Sprr2f* mRNA expression, and (H) histological score at day 2 post renal IR injury in 10-week-old male mice. (I) Experimental group (left) and correlation between preoperative serum IGF-1 concentration and urea levels (right), (J) serum creatinine (K), *Krt20* and *Sprr2f* mRNA expression, and (L) histological score; at day 2 post renal IR injury in 10-week-old female mice. (M) Experimental group (left) and correlation between preoperative serum IGF-1 concentration and urea levels (right), (N) serum creatinine, (O) *Krt20* and *sprr2f* mRNA expression, and (P) histological score at day 2 post renal IR injury in 18-month-old male mice. ∗*p* values for B-D were calculated with one-way ANOVA followed by a Tukey's post hoc analysis, and for E-P with an F-test to compare the linear regression model against the null hypothesis, (∗∗∗*p* < 0.001 ∗∗∗∗*p* < 0.0001), *p* values for B < 0.0001, = 0.001 and <0.0001, for C = 0.0002, <0.0001 and <0.0001, and for D < 0.0001, <0.0001 and <0.0001. Sample sizes: (B-D), *n* = 6 for all conditions for 10-week-old C57BL/6J male mice; (I–L), *n* = 8 for all conditions for 10-week-old female C57BL/6J mice; (M-P), *n* = 8 for all conditions in 18-month-old C57BL/6J male mice. Data are shown as mean ± SD. See also Extended Data Figure S1.

10-week-old C57BL/6J male mice were fed *ad libitum* for one week with semi-purified diets containing varying protein levels, from 19.8% (control) down to 6.4% (low protein) of energy. Further protein dilution was achieved using a carbohydrate-loading drink, which induced a voluntary 2 to 3-fold reduction in solid food intake, as previously published.^18^ Mice were separated into four groups based on their protein intake. 1. Control (CTRL) with 19.8% protein. 2. Low protein (LP) with 6.4% protein. 3. Control diet with a high carbohydrate (HC) drink resulting in a mean protein intake of 3.3%. 4. Low protein diet + high carbohydrate drink (LPHC) resulting in a mean protein intake of 1.7%. First, we utilized the geometric framework^16^ to evaluate the effects of different macronutrients on serum IGF-1. Serum IGF-1 was most robustly associated with protein intake, and IGF-1 levels were minimally influenced by fat and carbohydrate intake (Figures 1A and S1A). Interestingly, the titration of protein intake produced a non-linear response on kidney (Figure 1B) and liver (Figure 1C) *Igf1* mRNA expression and serum IGF-1 concentration (Figure 1D, 48.65 ± 16.47 *versus* 15.55 ± 3.19 *versus* 32.63 ± 16.85 *versus* 8.78 ± 3.61 ng/mL, in 19.8%, 6.4%, 3.3% and 1.7% respectively) with 6.4%, 3.3% and 1.7% of protein intake leading to similar IGF-1 downregulation compared to the 19.8% protein intake (CTRL) group. Having demonstrated a correlation between protein intake and IGF-1, we next tested the association with IR injury. After one week of dietary preconditioning, mice underwent a right nephrectomy and unilateral left kidney IR injury as previously described.^18^ The four different diets did not have any significant impact on pre-surgery serum urea and creatinine levels (Figures S1B and S1C). All groups returned to a control 19.8% protein *ad libitum* regimen immediately postoperatively. In this context, preoperative serum IGF-1 concentration was positively correlated with serum urea (Figure 1E, R^2^ = 0.43, *p* = 0.0017) and creatinine (Figure 1F, R^2^ = 0.38, *p* = 0.004) levels indicating impaired renal function, with *Krt20* (Figure 1G, R^2^ = 0.27, *p* = 0.0223) and *Sprr2f* (Figure 1G, R^2^ = 0.40, *p* = 0.0028) mRNA expression, which are biomarkers of tubular damage,^28^ and with histological damages as assessed by our modified version of the Goujon score^29^ (Figures 1H and S1D, R^2^ = 0.32, *p* = 0.0043).

Previous studies reported sex specific sensitivity to PR as well as IR injury.^30^^,^^31^^,^^32^^,^^33^ Thus, to substantiate our findings, 10-week-old C57BL/6J female mice were subjected to voluntary PR through exposure to HC drink resulting in a mean protein intake of 6.3% followed by renal IR injury. This regimen was chosen due to its ability to lower IGF-1 without any calory restriction, which might be more clinically relevant, especially for older patients who are at risk of sarcopenia as previously demonstrated.^18^^,^^34^ Of note, the duration of ischemia was increased to 34 min to reach measurable damages (Figure 1I). Baseline IGF-1 serum concentration was similar between males and females (Figure S1E). In accordance with our observations in males, females serum IGF-1 levels were positively correlated with renal damage, assessed by serum urea (Figure 1I, R^2^ = 0.57, *p* = 0.0049) and creatinine (Figure 1J, R^2^ = 0.34, *p* = 0.0011) levels, *Krt20* (Figure 1K, R^2^ = 0.48, *p* = 0.0014) and *Sprr2f* (Figure 1K, R^2^ = 0.21, *p* = 0.0147) mRNA expression and histological injury (Figures 1L and S1D, R^2^ = 0.27, *p* = 0.0025).

IGF-1 levels were also shown to decrease with age in both humans and animal models.^35^ Thus, 18-months old C57BL/6J male mice underwent one-week PR through exposure to HC drink resulting in a mean protein intake of 5.2% followed by renal IR injury as above. Old mice are known to be more sensitive to ischemia.^36^ The ischemia duration was thus reduced to 12 min (Figure 1M). Consistent with the literature, baseline serum IGF-1 concentration was significantly lower in aged mice than in their younger counterparts (Figure S1E). Consistent with our previous findings, following IR injury, serum IGF-1 levels in aged mice were positively correlated with serum urea (Figure 1M, R^2^ = 0.1962, *p* = 0.0357) and creatinine (Figure 1N, R^2^ = 0.0004, *p* = 0.0554) levels, *Krt20* (Figure 1O, R^2^ = 0.2891, *p* = 0.7819) and *Sprr2f* (Figure 1O, R^2^ = 0.0309, *p* = 0.1941) mRNA expression and histological damages (Figures 1P and S1D, R^2^ = 0.0452, *p* = 0.0002). Of interest, taken together, these data suggest that higher IGF-1 levels are associated with IR injury independently of sex and age.

### Exogenous insulin-like growth factor-1 increases ischemia-reperfusion injury

**Figure 2:**
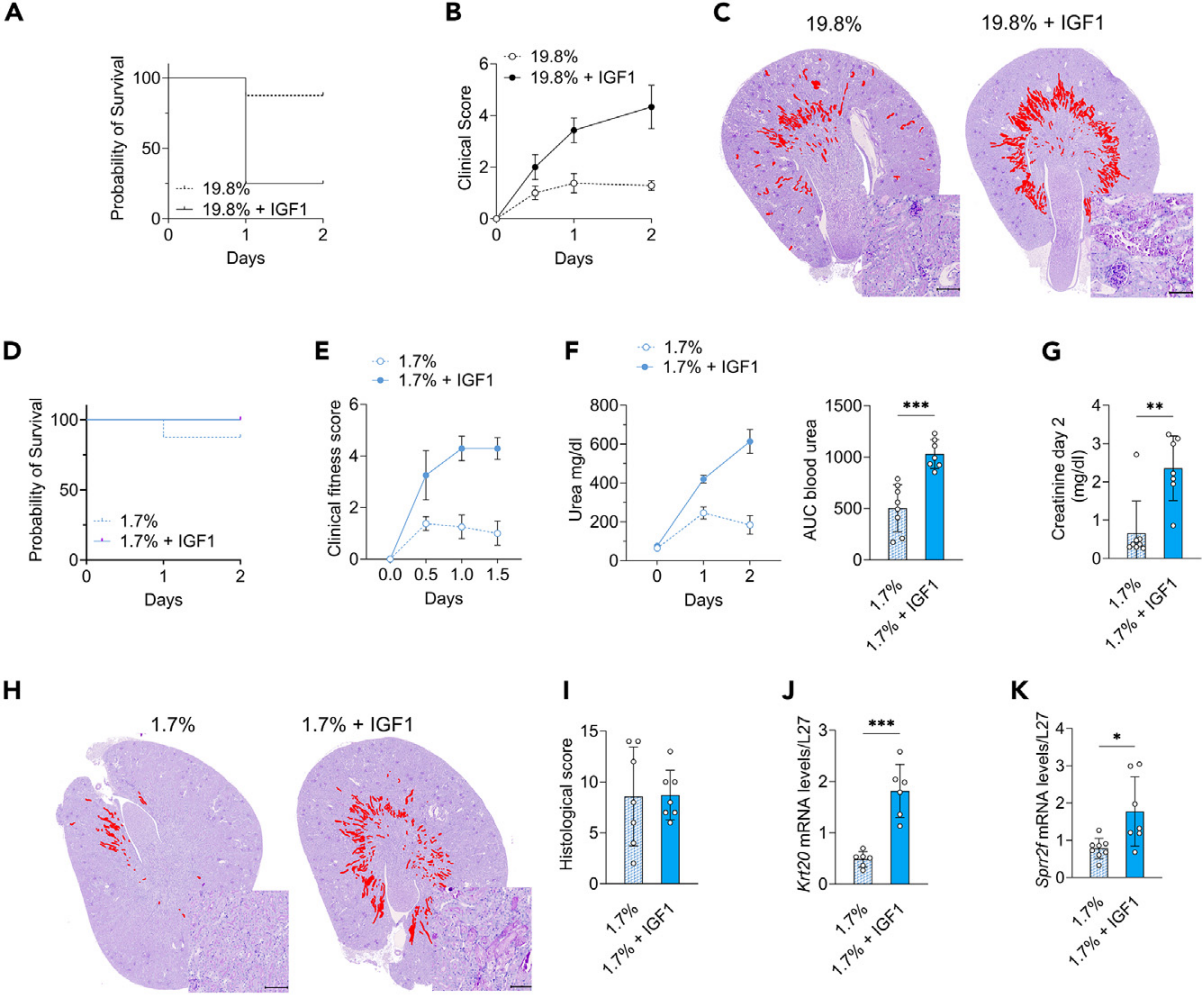
IGF-1 increases the severity of ischemia-reperfusion injury (A) Kaplan-Meier survival curve and (B) Clinical score at the indicated time after renal IR injury in 10-week-old male mice exposed to one week CTRL 19.8% protein diet and treatment with vehicle or rhIGF-1 via osmotic pumps. (C) Representative cross-sections of PAS-stained kidneys with areas of tubulointerstitial necrosis digitally highlighted in red (×10 magnification; scale bar 100μm) at day 2 post renal IR injury in 10-week-old male mice exposed to one week CTRL 19.8% protein diet and treatment with vehicle or rhIGF-1 via osmotic pumps. (D) Kaplan-Meier survival curve, (E) clinical score (F) urea over time (left) and AUC (right) in 10-week-old male mice exposed to one-week LPHC 1.7% protein diet and treatment with vehicle or rhIGF-1 via osmotic pumps. (G) Serum creatinine concentration, (H) Representative cross sections of PAS-stained kidneys with areas of tubulointerstitial necrosis digitally highlighted in red (×10 magnification; scale bar 100μm), (I) Histological score, (J) Kidney relative *Krt20* and (K) *Sprr2f* mRNA at day 2 post renal IR injury in 10-week-old male mice exposed to one-week LPHC 1.7% protein diet and treatment with vehicle or rhIGF-1 via osmotic pumps. ∗*p* values for F-G and I–K were calculated with unpaired two-tailed t-test, ∗*p* < 0.05 ∗∗*p* < 0.01 ∗∗∗*p* < 0.001. *p* values for F = 0.0001, for G = 0.0018, for I = 0.9457, for J = 0.0001, and for K = 0.0126. Sample sizes: *n* = 8 for all conditions for 10-week-old C57BL/6J male mice. For B, E and F (left), data are shown as mean ± SEM; for F (right)-J and I-K, data are shown as mean ± SD. See also Extended Data Figure S2.

Having demonstrated that serum IGF-1 was correlated with injury following kidney IR, we sought to investigate the effect of IGF-1 supplementation. Recombinant human IGF-1 (rhIGF-1) at 4 mg/kg/day or control sodium chloride vehicle (Veh) was administered to 10-week-old C57BL/6J male mice via osmotic minipumps for one week, prior to renal IR injury. IGF-1 administration did not alter food and water intake (Figures S2A and S2B) but significantly increased body weight (Figure S2C). As expected, human (h)IGF-1 was elevated, with a subsequent reduction of endogenous mouse (m)IGF1 in mice treated with high dose of rhIGF-1 (Figures S2D and S2E). In the kidney, rhIGF-1 administration resulted in increased phosphorylation of AKT on Serine 473, consistent with the stimulation of IGF-1R downstream signaling^37^ (Figure S2F). Exogeneous IGF-1 administration also resulted in the reduction of blood glucose (Figure S2G), serum triglycerides (Figure S2H) and insulin concentration (Figure S2I). Preoperative IGF-1 administration reduced survival after kidney IR injury, with 75% mortality at 2 days after surgery compared to 20% mortality in the vehicle treated group (Figure 2A). Consistently, clinical wellbeing was reduced in IGF-1 treated mice (Figure 2B). This correlated with more severe histological tubulointerstitial damages (Figure 2C). Of note, renal function could not be reliably assessed due to the excess mortality observed with IGF-1 treatment.

Given that PR reduces IR injury and serum IGF-1 levels, we tested whether exogenous IGF-1 would abrogate PR benefits. Mice on 1.7% protein regimen (achieved via the combination of carbohydrate loading and *ad libitum* feeding of a diet containing 6.4% of protein) were treated with rhIGF-1 or without (vehicle) for 1 week prior to IR injury as above. Exogenous IGF-1 did not impact food and water intake (Figures S2J and S2K) nor changed body weight (Figure S2L). Similar to mice on a control diet, rhIGF-1 administration increased AKT phosphorylation on Serine 473 (Figure S2M), decreased serum mIGF-1 (Figure S2N), and induced a further reduction of blood glucose (Figure S2O) serum triglycerides (Figure S2P) and insulin concentration (Figure S2Q). Survival was similar in mice treated with rhIGF-1 (Figure 2D), but clinical wellbeing was reduced in mice treated with IGF-1 (Figure 2E). Consistently, exogeneous IGF-1 administration abrogated PR mediated protection from IR injury, as demonstrated by increased day 2 postoperative serum urea (Figure 2F, 614 ± 161 versus 184 ± 134 mg/dL) and creatinine levels (Figure 2G, 0.67 ± 0.83 versus 2.35 ± 0.84 mg/dL), tubulointerstitial damages and tubular necrosis (Figures 2H and 2I) as well as *Krt20* and *Sprr2f* gene expression (Figures 2J and 2K). Taken together, these data demonstrate that exogenous IGF-1 supplementation is sufficient to increase renal IR injury and to abrogate PR benefits. Targeting IGF-1 or the IGF-1 signaling pathway may therefore be used as a therapy to reduce IR injury.

### Blockade of insulin-like growth factor-1 signaling reduces ischemia-reperfusion injury

**Figure 3:**
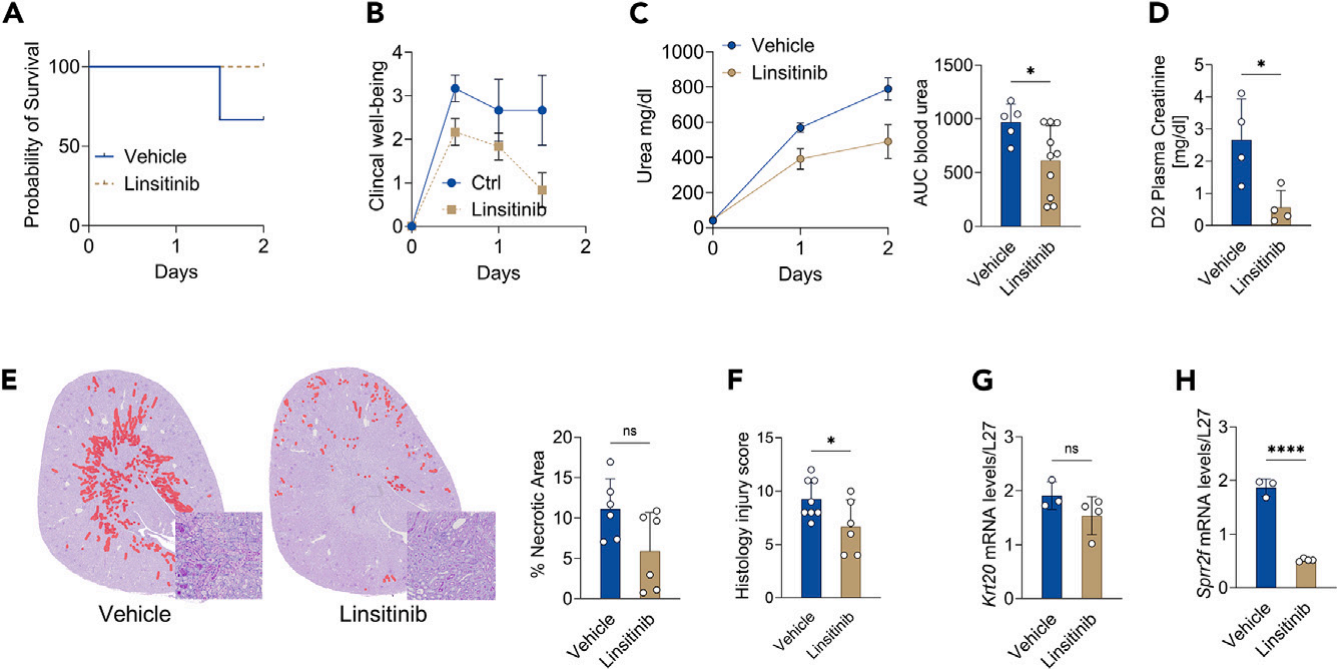
Blockade of IGF1 signaling reduces ischemia-reperfusion injury (A) Kaplan-Meier survival curve, (B) Clinical score (C) urea over time (left) and AUC (right) in 10-week-old male mice treated with vehicle or linsitinib (D) Serum creatinine concentration, (E) Representative cross sections of PAS-stained kidneys with areas of tubulointerstitial necrosis digitally highlighted in red (left; ×10 magnification; scale bar 100μm) and quantification (right), (F) Histological score, (G) Kidney relative *Krt20* and (H) *Sprr2f* mRNA at day 2 post renal IR injury in 10-week-old male mice treated with vehicle or linsitinib ∗*p* values for (C–H) were calculated with unpaired two-tailed T-test, ∗*p* < 0.05 ∗∗∗∗*p* < 0.0001. *p* values for C = 0.0383, for D = 0.0220, for E = 0.0626, for F = 0.0450, for G = 0.1841, and for H < 0.0001. Sample sizes: *n* = 8 for all conditions for 10-week-old male C57BL/6J mice. For B and C (left), data are shown as mean ± SEM; for C (right)-H, data are shown as mean ± SD. See also Extended Data Figure S3.

We next asked whether IGF-1 signaling inhibition alone could reduce IR injury. Mice were treated with 20 mg/kg of the IGF-1R/Insulin Receptor Kinase Inhibitor linsitinib (OSI-906)^38^ once a day for 3 days before right nephrectomy and contralateral IR injury.^18^ Linsitinib administration did not affect food or water intake (Figures S3A and S3B) but was associated with a reduction in body weight (Figure S3C). Linsitinib resulted in an increase in blood glucose (Figure S3D) without affecting mIGF1 levels (Figure S3E). As expected,^37^ renal AKT phosphorylation on Serine 473 was inhibited by linsitinib (Figure S3F). Importantly, linsitinib improved survival after kidney IR injury, with 0% mortality at 2 days post-surgery compared to 33% mortality in the vehicle treated mice on a regular diet (Figure 3A). Postoperative clinical wellbeing was also improved in linsitinib treated mice (Figure 3B). Furthermore, mice treated with linsitinib had lower day 2 postoperative serum urea (Figure 3C, 490 ± 318 versus 789 ± 139 mg/dL) and creatinine levels (Figure 3D, 0.56 ± 0.53 versus 2.67 ± 1.26 mg/dL), histological tubulointerstitial damages tubular necrosis (Figures 3E and 3F) and *Krt20* and *Sprr2f* gene expression (Figures 3G and 3H). Taken together, these data suggest that preoperative IGF-1 signaling inhibition is sufficient to protect against renal IR injury independently of any dietary interventions.

### Identification of genes regulated by Insulin-Like Growth Factor-1 associated with protection from ischemia-reperfusion injury

We finally sought to understand the changes in gene expression induced by both endogenous and exogenous IGF-1, and how they underlie the protective effects on IR injury.

**Figure 4:**
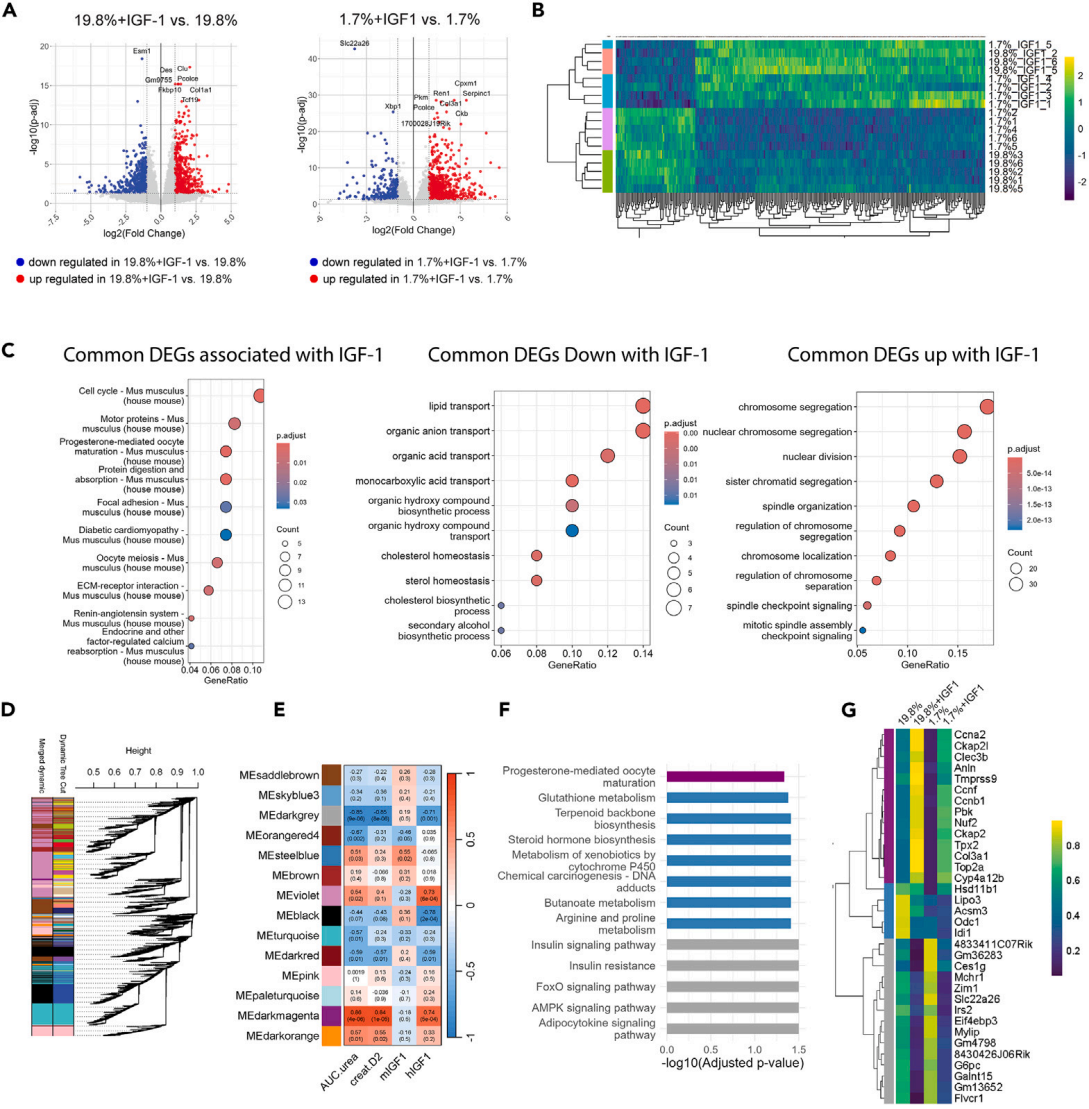
Identification of gene and modules regulated by IGF-1 in the kidney that are associated with protection from ischemia-reperfusion injury (A) Volcano plot of differentially expressed genes down (blue) and up-regulated (red) in 19.8%+IGF1 as compared to 19.8% (left) and in 1.7%_IGF1 as compared to 1.7% (right) in preconditioned mice. (B) Heatmap of log2CPM expression levels of the common DEGs in kidney with gene set enrichment analysis. Yellow indicates high expression and blue indicates low expression. (C) Top 10 KEGG pathways from common DEGs (ranked by *p*-value) (Left) associated with IGF-1 signature, (Middle) down- and (Right) up-regulated following IGF-1 administration. Gene ratio (x axis) is the percentage of significant genes over the total genes in a given pathway. (D) Dendrogram of clusters displaying gene expression similarity among samples. Merge cluster dynamic tree cut algorithm identifies distinct color-coded modules at the bottom. (E) Module-Trait relationships with serum urea, creatinine, mIGF-1 and hIGF-1 levels identified through Weighted Gene Co-expression Network Analysis (WGCNA) from kidneys of male mice in the indicated group. The top line corresponds to Pearson R^2^ and bottom line to adjusted *p* value. Color scales represent positive correlation (red) and negative correlation (blue). (F) KEGG enriched pathways from common DEGs in each module significantly correlated modules with serum urea and hIGF-1. (G) Heatmap of z-transformed expression levels of common DEGs in each module with significant Module-Trait relationships with serum urea and hIGF1. Yellow indicates high expression and blue indicates low expression.∗*p* values for (A and B) were calculated with Wald parametric test and for E with Pearson correlation. Sample sizes: n = 3–5 10-week-old C57BL/6J male mice, *n* = 5 for 19.8%, *n* = 3 for 19.8%+IGF1, *n* = 5 for 1.7%, and *n* = 5 for 1.7%+IGF1. See also Extended Data Figure S4.

10-week-old C57BL/6J male mice were preconditioned for one week with 19.8% or 1.7% protein regimen with or without exogenous rhIGF-1 administration prior to renal IR. We chose the LPHC 1.7% protein regimen as PR because it is the most extreme PR regimen we use and is therefore more likely to show clear difference regarding the impact of PR on IR injury in our model. Bulk mRNA sequencing was performed on the baseline kidneys (retrieved by nephrectomy) based on the assumption that gene changes and potential targets, associated with higher IGF-1 pre-injury will impact sensitivity to IR.^19^^,^^39^ Using Wald parametric test, we identified the most common differentially expressed genes (DEGs) in the kidney of mice exposed to 19.8% versus 19.8%+IGF1 and 1.7% versus 1.7%+IGF1 (Figure 4A). Gene set enrichment analysis identified 343 common genes significantly associated with IGF-1 supplementation as compared to the group with the same diet but without IGF-1 (Figure 4B). Using Kyoto Encyclopedia of Genes and Genomes (KEGG), we found that IGF-1 signature was associated with cell cycle, protein digestion and absorption as well as extracellular matrix interaction (Figure 4C). Pathways related to fat and carboxylic acids metabolisms such as lipid, organic anion, organic acid, monocarboxylic acid and organic hydroxy compound transports, organic hydroxy compound and secondary biosynthetic processes, cholesterol and sterol homeostasis were down-regulated in response to exogenous IGF-1 treatment (Figure 4D). On the other hand, proliferation and mitosis related pathways such as chromosome, nuclear chromosome and sister chromatid segregation, nuclear division and spindle checkpoint signaling were up-regulated (Figure 4D). To identify the genes which correlated with serum IGF-1 and serum urea, reflective of renal IR injury we aggregated and analyzed all expressed protein-coding genes (*n* = 16128) using weighted correlation network analysis (WGCNA).^18^^,^^40^ A total of 14 WGCNA modules were identified (Figure 4D), with each WGCNA module containing 104 to 4678 genes. The darkgrey module negatively correlated (*p* < 0.05) with post-operative serum urea and creatinine levels and with exogenous hIGF-1 serum levels. The steelblue module positively correlated (*p* < 0.05) with postoperative serum urea and with endogenous mIGF-1 serum levels. The darkmagenta module positively correlated (*p* < 0.05) with post-operative serum urea and creatinine levels and exogenous hIGF-1 serum levels (Figure 4E). Genes in these modules significantly associated (R^2^ > 0.5, *p* < 0.05) with serum urea and creatinine levels as well as IGF-1 supplementation (higher hIGF-1, lower mIGF-1) were involved in “progesterone mediated oocyte maturation,” previously linked to IGF downstream signaling via the PI3K/AKT/mammalian target of rapamycin complex (mTORC)-1 pathway^41^^,^^42^ (Figures 4F and S4A). Genes significantly associated (R2 > 0.5, *p* < 0.05) with serum urea but inversely correlated with IGF-1 supplementation (lower hIGF-1 and higher mIGF-1) were involved in metabolic and antioxidant processes such as ”glutathione metabolism,” “terpenoid backbone biosynthesis,” Steroid hormone biosynthesis,” “metabolism of xenobiotics by cytochrome P450,” chemical carcinogenesis – DNA adducts,” “butanoate metabolism” and “arginine and proline metabolism” (Figures 4F and S4B). Genes inversely associated (R2 > 0.5, *p* < 0.05) with serum urea, creatinine, and IGF-1 supplementation (higher hIGF-1, lower mIGF-1) were involved in inflammatory and insulin sensing pathways such as ”insulin signaling pathway,” “Insulin resistance,” “FoxO signaling pathway,” “AMPK signaling pathway,” and “adipocytokine signaling pathway” (Figures 4F, 4G, and S4C). Altogether, these data suggest that downstream IGF-1, insulin sensing and inflammatory pathways such as FoxO and AMPK signaling pathways might mediate sensitivity to renal IR injury.

## Discussion

In this study, we demonstrated that one week PR in mice reduces circulating IGF-1 levels. IGF-1 positively correlated with increased damages following renal IR injury independently of sex and age. IGF-1 supplementation increased renal IR injury and abrogated PR benefits. Importantly, IGF-1R inhibition was sufficient to promote resistance against renal IR injury which could be of interest in the context of solid organ transplantation, vascular surgery, or myocardial infarction.

Here we demonstrate that targeting the IGF-1 pathway could serve as a novel treatment against renal IR injury. Importantly protection from IR injury was achieved in mice treated with IGF-1R inhibitor for only 3 days. Consistently, IGF-1 and IGF-1R inhibition were shown to reduce inflammation in a mouse model of diabetic kidney disease^43^^,^^44^ and as a potential anti-aging treatment.^45^ Several inhibitors of the IGF-1R are already used in clinical trials.^46^ Linsitinib is FDA-approved for severe thyroid eye disease (NCT05276063), and cancer (NCT01533246,^47^ NCT00889382,^48^ NCT02546544^49^ and NCT01560260^50^). Linsitinib is a dual inhibitor of IGF-1R and insulin receptor which brings concerns for potential side effects related to the induction of hyperglycemia, which was however only observed in less than 1% of patients.^48^^,^^50^^,^^51^ Of note, IGF-1 inhibition might be contraindicated in patients with diabetes as IGF-1 enhances insulin sensitivity by promoting glucose uptake and utilization.^52^^,^^53^ In the above-mentioned clinical trials (thyroid eye disease, prostate and ovarian cancers, Ewing sarcomas and gastrointestinal stromal tumors), linsitinib was administered for durations ranging from twice a day to once every three days for three to four weeks.^48^^,^^50^^,^^51^ The optimal timing and duration of therapeutic IGF-1R inhibition still need to be investigated further. Voluntary adherence to a PR has proven to be challenging. Pharmacological inhibition of IGF-1 or IGF-1R signaling could therefore be an alternative to dietary intervention. Additionally, it could be used in population where PR is contraindicated or to promote healthy aging.

Interestingly, IGF-1 was associated with increased IR injury independently of the sex. On the other hand, IGF-1 effects on aging and stress resistance have been found to be sex dependent. This could be attributed to a sex-differential regulation of oxidative balance and immune response.^54^^,^^55^ Indeed, sex differential regulations of Nuclear factor erythroid-derived 2-like (NRF)-2, mTOR and FoxO3 have been reported, which are all part of the IGF-1 downstream pathway.^23^^,^^56^

Using kidney total mRNA sequencing, we observed a downregulation of glutathione metabolism upon rhIGF-1 supplementation. This is opposite to the proposed IGF-1 antioxidant effect via increasing glutathione peroxidase activity *in vitro.*^57^ rhIGF-1 supplementation increased progesterone signaling, which was linked to IGF-1R activation via PI3K/AKT/mTORC-1.^41^^,^^42^ Interestingly, progesterone was reported to have sex-specific effects on kidney and brain IR injury by exerting a protective effect on female mice and having rather a negative impact on male mice notably by increasing mitochondrial fragmentation following IR injury.^58^ *In vitro*, incubation of astrocytes from male rats with progesterone decreased viability and increased apoptosis.^59^ We also observed a significant downregulation of FoxO signaling pathway following rhIGF-1 supplementation. IGF-1 is known to inhibit FoxO via the activation of the PI3K/AKT pathway.^60^ Of interest, FoxO reduced hepatic IR injury via the reduction of oxidative stress and apoptosis.^61^^,^^62^ Additionally, we also observed a downregulation of AMPK signaling pathway. IGF-1 was previously demonstrated to reduce AMPK via mTORC-1.^63^ Consistently, AMPK activation reduced IR injury of the heart, brain, kidney, liver, lung and intestine via its regulation of energy metabolism, oxidative stress, mitochondrial function, autophagy and inflammatory responses.^64^ In the kidney, AMPK activation reduced apoptosis following IR injury.^65^

IGF-1 also reduced insulin resistance in our model confirming the results of previous studies.^11^ IGF-1 and insulin actions might be difficult to separate from one another and have paradoxical effects.^66^^,^^67^ Increased insulin action promoted cell survival after an ischemic insult through increased insulin/PI3K/AKT signaling in heart^68^^,^^69^^,^^70^ and hepatic IR^71^ via reduced apoptosis.^72^ It was also reported that the reduction of insulin signaling extends longevity and increases stress resistance in part through the activation of cytoprotective gene expression normally inhibited by insulin/AKT signaling.^66^^,^^67^^,^^73^ IGF-1 can activate the insulin receptor directly. Thus, whether IGF-1 protective or harmful actions depend on insulin, IGF-1 or a combination of both remains to be established. Finally, targeting FoxO and AMPK signaling might be an alternative solution to reduce IR injury in patients that could not tolerate PR or IGF-1R blockade.

It was also demonstrated that short-term exogenous IGF-1 treatment improved resilience in mice and rats models of kidney, heart and brain IR injury.^24^^,^^25^^,^^26^^,^^27^ In these models rhIGF-1 was administered immediately before or after the interventions for a maximum duration of 4 days. How can both increased and decreased IGF-1 signaling be beneficial? In the context of acute stress resistance, as long as both of these mechanisms are separated in time relative to the acute stress, they are not necessarily mutually exclusive: reduced IGF-1 signaling during the dietary preconditioning period may increase FoxO-dependent antioxidant gene expression, while increased antiapoptotic IGF-1 signaling after injury may prevent cell death as seen with insulin.

We also recently demonstrated that fibroblast growth factor (FGF)-21 was required and sufficient for PR benefits.^18^ FGF-21 was shown to have a direct impact on IGF-1 concentration and these two protective mechanisms are therefore likely interconnected. Indeed, FGF-21 reduces concentrations of the active form of signal transducer and activator of transcription 5 (STAT5), a key mediator of growth hormone (GH) actions, leading to decreased IGF-1 expression. Additionally, FGF-21 increases the expression of IGF-1 binding protein-1 and of suppressor of cytokine signaling 2, which further reduce IGF-1 activity.^74^^,^^75^^,^^76^ Our previous results also demonstrated that the hydrogen sulfide (H_2_S) producing enzyme cystathionine γ-lyase (CGL) is required for the benefits of dietary restriction, including protection from ischemia^19^ and IR injury.^39^ Interestingly, H_2_S was shown to induce S-sulfhydration of IGF-1R thus reducing its activity.^77^ Whether IGF-1 inhibition during PR requires H_2_S is unknown.

Short-term PR might be an effective alternative to modulate endogenous IGF-1 levels and reduce IR injuries. Here we showed that a reduction from 19.8% to 6.4% protein intake was sufficient to reduce serum IGF-1 concentration 3-folds. The threshold of protein intake necessary to obtain this significant reduction in IGF-1 and protection from IR might be different in human, which will need to be determined.

In conclusion, in mice, one week PR reduces IGF-1 and IGF-1R inhibition is sufficient to increase resilience against kidney IR injury through various mechanisms which may include decreased insulin signaling and increased FoxO and AMPK signaling pathways. FDA approved IGF-1/IGF-1R inhibitors such as linsitinib could be rapidly tested in clinical trials to reduce renal IR injury in human.

### Limitations of the study

The study has two key limitations: (1) Linsitinib is a dual inhibitor of both IGF-1R and the insulin receptor, making it difficult to disentangle the actions of IGF-1 from insulin. (2) The impact of IGF-1 on ischemia-reperfusion injury may be time-dependent. Reduced IGF-1 signaling during dietary preconditioning could enhance FoxO-mediated antioxidant gene expression, while post-injury IGF-1 signaling might prevent cell death through its antiapoptotic effects. The optimal timing and duration of IGF-1R inhibition, especially in human patients, requires further investigation.

## Resource availability

### Lead contact

Requests for further information and resources should be directed to and will be fulfilled by the lead contact, Alban Longchamp, M.D., Ph.D. (alongchamp@mgh.harvard.edu).

### Materials availability

This study did not generate new materials.

### Data and code availability

• All data related to the bulk RNA sequencing analysis are available on Github.com at https://github.com/Longchamp-Lab/lyon-agius-et-al-iScience-2024 and on Figshare at https://doi.org/10.6084/m9.figshare.27180369. All other data generated or analyzed during this study are included in the article and supplementary tables and figures.
• All custom scripts are available on Github.com at https://github.com/Longchamp-Lab/lyon-agius-et-al-iScience-2024 and on Figshare at https://doi.org/10.6084/m9.figshare.27180369.
• All other relevant data are available from the corresponding author on request.

## Acknowledgments

We thank our deeply regretted friend and mentor, the late James R. Mitchell. This work was supported by the 10.13039/501100001711Swiss National Science Foundation to AL (SNSFPZ00P3-185927) and to Aly (SNSF323530-221859), the Mercier Foundation to AL, the Mendez National Institute of Transplantation Foundation to AL, the Novartis foundation to AL and FA, the Leenaards Foundation to AL, the University of Lausanne (projet de recherche interdisciplinaire) to DG and AL, the Fondation pour la recherche en chirurgie thoracique et vasculaire to FA, SD and AL, the Union des Sociétés Suisses des Maladies Vasculaires to SD and Fondation Medi-CAL Futur to DG. We are grateful to the Mouse Pathology Facility (MPF) and the Cellular Imaging Facility (CIF) of the University of Lausanne for their support and expertise.

## Author contributions

Aly, TA, MRM, KK, LS, SM, FA, SD, DG, and AL participated in research design. Aly, TA, MRM, SM, FA, SD, TK, LR, KU, HY, DG, and AL participated in the writing of the article. Aly, TA, MRM, KK, LS, SM, FA, DG, and AL participated in the performance of the research. MRM and SM contributed new reagents or analytic tools. Aly, TA, MRM, KK, LS, SM, FA, SD, DG, and AL participated in data analysis. Aly, FA, SD, DG and AL obtained funding.

## Declaration of interests

The authors declare no competing interests.

## STAR★Methods

### Key resources table

**Table:** 

REAGENT or RESOURCE	SOURCE	IDENTIFIER
**Antibodies**		

Protein Kinase B (Akt) antibody	Cell signaling	Cat# 9272; RRID: AB_329827
pAKT Ser 473	Cell signaling	Cat# 4060; RRID: AB_2315049

**Chemicals, peptides, and recombinant proteins**		

Recombinant human IGF-1	Peprotech	Cat# 100-11
Linsitinib (OSI-906)	Med Chem Express)	Cat# HY-10191
Passive Lysis Buffer	Promega	Cat# E1941

**Critical commercial assays**		

Pierce™ Reversible Protein Stain Kit for PVDF Membranes	ThermoFisher Scientific	Cat# 24585
Triglyceride kit using a kit	Sigma-Aldrich	Cat# TR0100
Mouse Creatinine Assay Kit	Crystal Chem INC	Cat# 80350
Mouse IGF-1 ELISA kit	Sigma-Aldrich	Cat# RAB0229
human IGF-1 ELISA kit	Sigma-Aldrich	Cat# RAB0228

**Deposited data**		

All data and script for Bulk RNAseq analysis	This paper	https://github.com/Longchamp-Lab/lyon-agius-et-al-iScience-2024

**Experimental models: Organisms/strains**		

Mus musculus C57BL/6J strain	Janvier lab	https://janvier-labs.com/fiche_produit/2-c57bl-6jrj/

**Software and algorithms**		

Zen Blue 3.4 software ().	Carl Zeiss	https://www.zeiss.com/microscopy/us/l/campaigns/software-overview.html?utm_source=google&utm_medium=cpm&utm_campaign=software_overview&gad_source=1&gclid=Cj0KCQjwpP63BhDYARIsAOQkATYEFI-a5Qw0YaZGD_XClaQMpSpItqsHX2geMaCWqoH55pt208-IIGkaAvzTEALw_wcB
ImageJ (v1.54r)	Fiji	https://imagej.net/ij/
QuantStudioTM 1.3 software	Thermofisher	https://www.thermofisher.com/us/en/home/life-science/pcr/real-time-pcr/real-time-pcr-instruments/quantstudio-systems.html?gclid=Cj0KCQjwpP63BhDYARIsAOQkATbUG4umqkfbDVP7DI0f1li3OujIe4DMLIN_DvJ7m1YUd0qFclhJth4aAg15EALw_wcB&ef_id=Cj0KCQjwpP63BhDYARIsAOQkATbUG4umqkfbDVP7DI0f1li3OujIe4DMLIN_DvJ7m1YUd0qFclhJth4aAg15EALw_wcB:G:s&s_kwcid=AL!3652!3!606132911240!e!!g!!quantstudio!17574808706!139287687298&cid=gsd_pcr_sbu_r02_co_cp1491_pjt9629_gsd00000_0se_gaw_rs_lgn_&gad_source=1
R Studio(v4.3.1).	Posit	
DESeq2 package in R (v1.40.2).	Michael Love	https://10.18129/B9.bioc.DESeq2
R package pheatmap (v1.0.12).	Raivo Kolde	https://10.32614/CRAN.package.pheatmap
R package ClusterProfiler (v4.8.3).	Guangchuang Yu	https://10.18129/B9.bioc.clusterProfiler
Prism 10.2	GraphPad Software	https://www.graphpad.com/
Biorender	Biorender	https://www.biorender.com/
org.Mm.e.g.,.db package in R (v3.17).	Marc Carlson	org.Mm.e.g.,.db package in R

### Experimental model and study participant details

#### Mice

All experiments were performed with the approval of the cantonal Veterinary Office (Service de la Consommation et des Affaires Vétérinaires SCAV-EXPANIM, authorization number VD3346, VD3554b, VD3768 and VD3816). All animal experimentation conformed to the Guide for the Care and Use of Laboratory Animals. 10–12 weeks old male and female, and 18-months-old male C57BL/6J mice (Janvier Labs, France) were used for all experiments. All mice were kept on *ad-libitum* (AL) access to food and tap water, and kept under standard housing conditions, with 12h light/12h dark cycles, 30–70% humidity and a temperature of 20°C–23°C unless specified otherwise.

#### Experimental diets

All experimental diets were based on diet 2125 from Granovit AG (Kaiseraugst, Switzerland), with 19.8% of calories from protein (hydrolyzed casein and individual crystalline amino acids), 10.4% from fat and 69.9% from carbohydrate. The low protein (LP) diet was custom prepared by Granovit AG based on diet 2125 with 6.4% protein/10.4% fat/83.2% carbohydrate (Granovit AG) and was provided *ad-libitum*. The nutritional value of the control diet was 3.6 kcal/gram, and 3.35 kcal/gram for the LP diet. The high sucrose (HC) drink was prepared by adding 50% sucrose (w/w) in the drinking water, giving 2 kcal of carbohydrate/gram of HC drink. HC drink was changed every two days. The mean intakes of each component of the diets in each group are detailed in Table S1.

#### Food and drink intake

Food pellets and bottles were weighed daily in all cages. The delta weight of food and drink was calculated, divided by the number of mice, and normalized by the weight of each individual mouse in the cage. Total calory intake was measured by calculating the sum of each macronutrient and their calories per gram (carbohydrates, proteins 4 kcal/gram, and fat 9 kcal/gram).

### Method details

#### Surgical models

##### Nephrectomy and renal ischemia-reperfusion injury

Mice were anesthetized with 3% isoflurane in 2L O_2_ and kept at 37°C with an electrical heating pad. Following a 2-cm abdominal incision, the vascular pedicles of the right and left kidneys were identified under a microscope. First, the right renal artery, vein, and ureter were ligated and cauterized. Immediately after, the right kidney was dissected and flash-frozen in liquid nitrogen. Second, the left pedicle was clamped for either 23 min for young male mice, 12 min for old male mice or for 34 min for young female mice with S&T vascular micro-clamps (FST 18055-03, Fine Science Tools). A darkening of the kidney was observed to ensure that the pedicle had been successfully clamped.

##### Postoperative care

The abdominal incision was sutured with 6-0 Prolene, and surgical staples were used to close the skin (427631, Aichele Medico AG). All mice received subcutaneous buprenorphine (0.05 mg/kg Temgesic, Reckitt Benckiser AG, Switzerland) every 10h following surgery. Additionally, they received paracetamol (2 mg/mL Dafalgan, UPSA) and buprenorphine (0.009 mg/mL Temgesic) in the drinking water for 48 h postoperatively. Mice clinical score included the following criteria: overall activity, posture and facial expression, grooming, presence of uncontrolled muscular contractions, balance and the presence of limping. Blood samples from the tail vein were taken pre-operatively, and at 24h postoperatively using a capillary tube. Serum was separated by centrifugation (2000 G for 20 min at 4°C) and flash frozen in liquid nitrogen before being stored at −80°C. On the second- or third day following surgery for renal IR injury, mice were euthanized under anesthesia via cervical dislocation, followed by exsanguination, and perfused with PBS. The remaining kidney was collected and cut in half transversally. One-half was flash-frozen in liquid nitrogen, and the other was fixed in 10% neutral buffered formalin and paraffin embedded for histology.

#### IGF-1 treatment

Recombinant human IGF-1 (Cat# 100-11, Peprotech) was dissolved and diluted in sterile distilled water to a final dosage of 4 mg/kg/day. The filled 1007D Alzet osmotic minipump was presoaked for 24h in NaCl at 37°C in a dry incubator. Mice were anesthetized as described. 1-cm incision was made in the skin of the upper back/neck to implant the sterile, preloaded minipump. 5-0 Prolene surgical thread was used to stitch the wound.

#### IGF1 receptor inhibition

20 mg/kg of linsitinib (OSI-906) (Cat# HY-10191, Med Chem Express) was injected intraperitoneally every morning for two days preceding the surgery and on the morning of the surgery 2h before operating. Glycemia was measured daily, 6h after the injection to control the effect of the treatment.

#### Histological analysis of kidneys

3-micron sections from paraffin-embedded half kidneys were stained with Periodic Acid Schiff (PAS) and paraffin-embedded livers with hematoxylin and eosin (H&E). The kidneys were scored histologically using a modified Goujon scoring method.^29^^,^^78^^,^^79^ This score was created to limit the observer’s subjectivity and to evaluate the entire section containing heterogeneous damage. The kidneys were imaged at a magnification of 20× using a Zeiss Axioscan Z.1 slide scanner (Carl Zeiss). The entire scanned section was analyzed using Zen Blue 3.4 software (Carl Zeiss) and scored on five parameters: 1) glomeruli integrity, 2) proximal tubule dilatation, 3) brush border integrity, 4) debris in the tubules, and 5) medulla integrity in the cortico-medullar area. Within each category, each item was graded on a scale of 0–3, with 0 representing “no damage” and 3 representing “extremely damaged.” Briefly, to assess glomerulus integrity, more than ten glomeruli were randomly selected from the section and assigned a score of 0–3. The same procedure was followed in the remaining categories. After that, the score for each category was converted to a percentage of damage. A final score between 0 and 5 was assigned based on this percentage of damage: 0 representing a percentage of damage between 0% and 15%; 1 for damage between 15% and 30%; 2 for damage between 30% and 45 percent; 3 for damage between 45 percent and 60%; 4 for damage greater than 60%; and 5 for damage greater than 75%. On a scale of 0–25, the final score was the sum of the scores for each category.

##### Necrotic area quantification

PAS-stained Entire scanned sections of kidneys were exported (50%) using Zen Blue 3.4 software (Carl Zeiss). Tubulointerstitial necrotic areas were identified using the following criteria: tubules with large debris/cast formation, large dilation, and tubular cell loss. The renal pelvis region has been excluded from quantification.

The necrotic area of digitally highlighted images was measured by calculating the highlighted area fraction using ImageJ (v1.54r) and defined as the necrotic area divided by the total kidney area.

#### Reverse transcription quantitative polymerase chain reaction (RT-qPCR)

Total RNA was isolated using the TriPureTM method (Roche, Switzerland) from 30 to 50mg of kidney, liver powder followed by complementary DNA (cDNA) synthesis using the Prime Script RT reagent kit (Takara). cDNA samples were loaded into a 384-well plate format (Applied Biosystems, ThermoFischer Scientific AG, Switzerland) using SYBR Green reagent–based PCR chemistry (10-mL reaction volume containing specific forward and reverse primers). The quantitative PCR program was run on a Viia 7 Real-Time PCR System, according to the manufacturer’s recommendations (Applied Biosystems, ThermoFischer Scientific AG, Switzerland). *Rpl27* was chosen as the housekeeping gene.^80^ Ct values for candidate and housekeeping genes were determined, and standard curves for each gene were calculated using serial dilutions. The relative standard curve method was used to determine the relative level of expression of genes. The primers listed in Table S2 were used, and analysis was performed using the QuantStudioTM 1.3 software (ThermoFischer Scientific AG, Switzerland).

#### Western blotting

Kidneys were flash-frozen in liquid nitrogen, grinded to power and resuspended in passive lysis buffer (Promega). Protein concentration was determined by DC protein assay (Bio-Rad Laboratories, Reinach, Switzerland). Approximately 10–20 μg of protein were loaded per well. Lysates were resolved by SDS-PAGE and transferred to a PVDF membrane (Immobilon-P, Millipore, Merck KGaA, Darmstadt, Germany) and blotted for AKT (Cat# 9272, Cell Signaling, 1:4000) and pAKT Ser 473 (Cat# 4060, Cell Signaling, 1:1000), and secondarily with HRP-conjugated anti-rabbit antibody (Dako). Membranes were revealed by enhanced chemiluminescence (Immobilon, Millipore) using the Azure 280 device (Azure Biosystems, Dublin, CA, USA) and analyzed using the Fiji (ImageJ 1.54r) software. Protein abundance was normalized to total protein using Pierce Reversible Protein Stain Kit for PVDF Membranes (Cat# 24585; ThermoFisher Scientific).

#### Blood analysis

Serum was isolated from blood taken from the tail vein pre-operatively and on days 1, 2, and 3 postoperatively by centrifugation (2000g for 20 min at 4°C). Triglyceride concentration was determined using a Sigma kit (Cat# TR0100). Glycemia was measured using a CONTOURXT glycemia reader. Urea was measured as published^12^ and using the Jung colorimetric method with reactive solution containing 100 mg/L *o*-phthalaldehyde (Cat# 32800, Serva), 300 mg/L N-(1-naphthyl) ethylenediamine dihydrochloride (Cat# 222488, Sigma), 2.5 mol/L sulfuric acid, 2.5 g/L boric acid, and 0.003% Brij 35. After 30 min in the dark and at room temperature, the response was measured at 505 nm with a Synergy Mx micro-plate reader (BioTek Instruments (Switzerland) GmbH). The serum creatinine level was determined using the mouse Creatinine Assay Kit (Cat# 80350, Crystal Chem INC). Mouse IGF-1 (Cat# RAB0229, Sigma-Aldrich) and human IGF-1 (Cat# RAB0228, Sigma-Aldrich) levels were determined using commercial ELISA kits using the manufacturer’s recommended protocol.

#### RNA-sequencing analysis

Data preprocessing, statistical computation, and visualization were performed using R (v4.3.1). Data preprocessing included filtering genes based on variance, expression across the samples, and missing values. Raw counts were normalized by performing a variance-stabilizing transformation using the DESeq2 package in R (v1.40.2). For gene-level testing and identification of differentially expressed genes (DEGs), statistical significance was assessed using deseq.wald statistical methods with contrasts: 19.8%+IGF1 vs. 19.8% and 1.7%+IGF1 vs. 1.7%. Only genes with an adjusted *p* value >0.05 and a log2 foldchange >1 were considered significant DEGs. Code is available on GitHub (https://github.com/orgs/Longchamp-Lab/).

##### Common DEGs analysis

Common DEGs from contrasts 19.8%+IGF-1 vs. 19.8% and 1.7%+IGF-1 vs. 1.7% were kept and 343 common genes were identified as IGF-1 driven signature. The clustered heatmap was generated using R package pheatmap (v1.0.12). Gene set enrichment analysis was performed using R package ClusterProfiler (v4.8.3). 16,128 detected genes were used as the enrichment background. Terms with a Benjamini-Hochberg adjusted *p* value <0.05 were considered significant.

##### WGCNA analysis

Raw counts were normalized by performing a variance-stabilizing transformation using the DESeq2 package in R (v1.40.2). Genes with counts <10 in more than 50% of samples were filtered out. The variance-stabilizing transformed gene expressions were subjected to WGCNA based on the WGCNA package in R (v1.72-1).^81^ WGCNA parameters were set as: no missing data expression; soft threshold = 5 (estimate value); adjacency = ”signed hybrid”; TOMType = ”signed”; merge cut height = 0.3. Function annotations of the genes were obtained using the org.Mm.e.g.,.db package in R (v3.17). The correlation of eigengenes with the external: AUC of serum urea and serum creatinine levels at day 2 post surgery, serum mouse IGF-1(mIGF1) and recombinant human IGF-1 (rhIGF1) at day 5 after start of the diet and treatment, was performed using ‘bicor’ biweight midcorrelation to obtain the most significant and robust associations. Intramodular analysis identified genes with high gene-module membership and gene-trait significance. The ClusterProfiler package in R (v4.8.3) was used for functional enrichment. *Z* score were calculated using z-transformation formula (x - mean(x))/sd(x). Common DEGs from each significantly associated module was included.

### Quantification and statistical analysis

All experiments adhered to the ARRIVE guidelines and followed strict randomization. All experiments and data analysis were conducted in a blind manner using coded tags rather than the actual group name. A power analysis was performed prior to the study to estimate sample-size. We hypothesized that HC preconditioning would reduce IR injury by 50%. Using an SD at ± 30% for the surgery and considering a power at 0.8, we calculated that *n* = 6 animals/group was necessary to validate a significant effect of the carbo-loading. Animals with pre-existing conditions (malocclusion, injury, abnormal weight) were not operated or excluded from the experiments upon discovery during dissection (kidney disease, tumor etc.). All *in vivo* experiments were analyzed using Prism 10.2 (GraphPad Software, USA). Data were presented as mean ± SD or as mean ± SEM and statistical significance was evaluated using Student’s t test, one- or two-way ANOVA, Wald parametric test and multiple comparisons were analyzed using Tukey’s and Sidak’s post-hoc tests. Correlation analyses were determined using linear regression or Pearson correlation. A *p*-value inferior or equal to 0.05 was defined as statistically significant. Artworks in Figure 1 have been created using BioRender (Academic License Terms, www.biorender.com).
