# Supplementary material for: Dietary or pharmacological inhibition of insulin-like growth factor-1 protects from renal ischemia-reperfusion injury in mice
Source: iScience. 2024 Oct 28;27(12):111256. doi: 10.1016/j.isci.2024.111256 (PMC11700642; doi:10.1016/j.isci.2024.111256)
Supplement: Document S1. Figures S1–S4, Tables S1, and S2 [file mmc1.pdf]

iScience, Volume *27*

Supplemental information

Dietary or pharmacological inhibition of insulin-like growth factor-1 protects from renal ischemia-reperfusion injury in mice

Arnaud Lyon, Thomas Agius, Michael R. Macarthur, Kevin Kiesworo, Louis Stavart, Florent Allagnat, Sarah J. Mitchell, Leonardo V. Riella, Korkut Uygun, Heidi Yeh, Sebastien Déglise, Déla Golshayan, and Alban Longchamp

SUPPLEMENTAL FIGURES AND LEGENDS

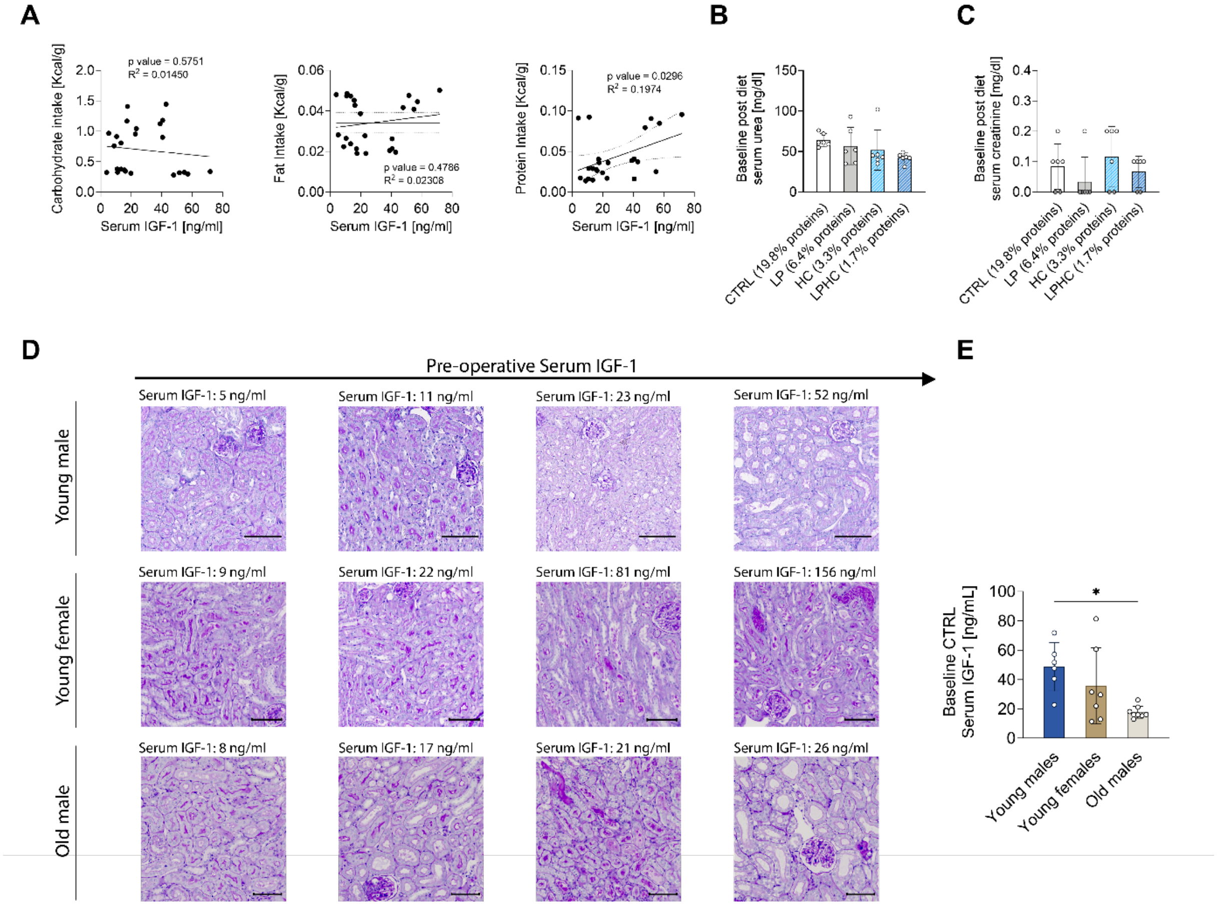

Figure S1. Additional information on the correlation between IGF-1 and sensitivity to ischemia-reperfusion injury.

(A) Correlation between preoperative carbohydrate intake, fat intake or protein intake and preoperative serum IGF-1 concentration in 10-week-old male mice.

(B) Post diet and preoperative serum urea and (C) creatinine levels in in 10-week-old male mice.

(D) Representative histology sections of PAS-stained kidneys (x10 magnification; scale bar 100µm) at day 2 post renal IR injury in 10-week-old male mice, 10-week-old female mice and 18-month-old male mice after one week exposure to protein restricted or control diet.

(E) Baseline IGF-1 serum concentration in 10-week-old male and female mice and 18-month-old mice on CTRL 19.8% proteins diet.

*p values for A were calculated with an F-test to compare the linear regression model against the null hypothesis. *$p < 0.05$. *p* value for E = 0.0134. Sample sizes: (A-C), n = 6 for all conditions for 10-week-old C57BL/6J male mice, (D), n = 6 for 10-week-old C57BL/6J male

mice, n = 8 for 10-week-old C57BL/6J female mice, n=8 for 18-month-hold C57BL/6J male mice. Data are shown as mean ± SD. See also Main Data Figure 1.

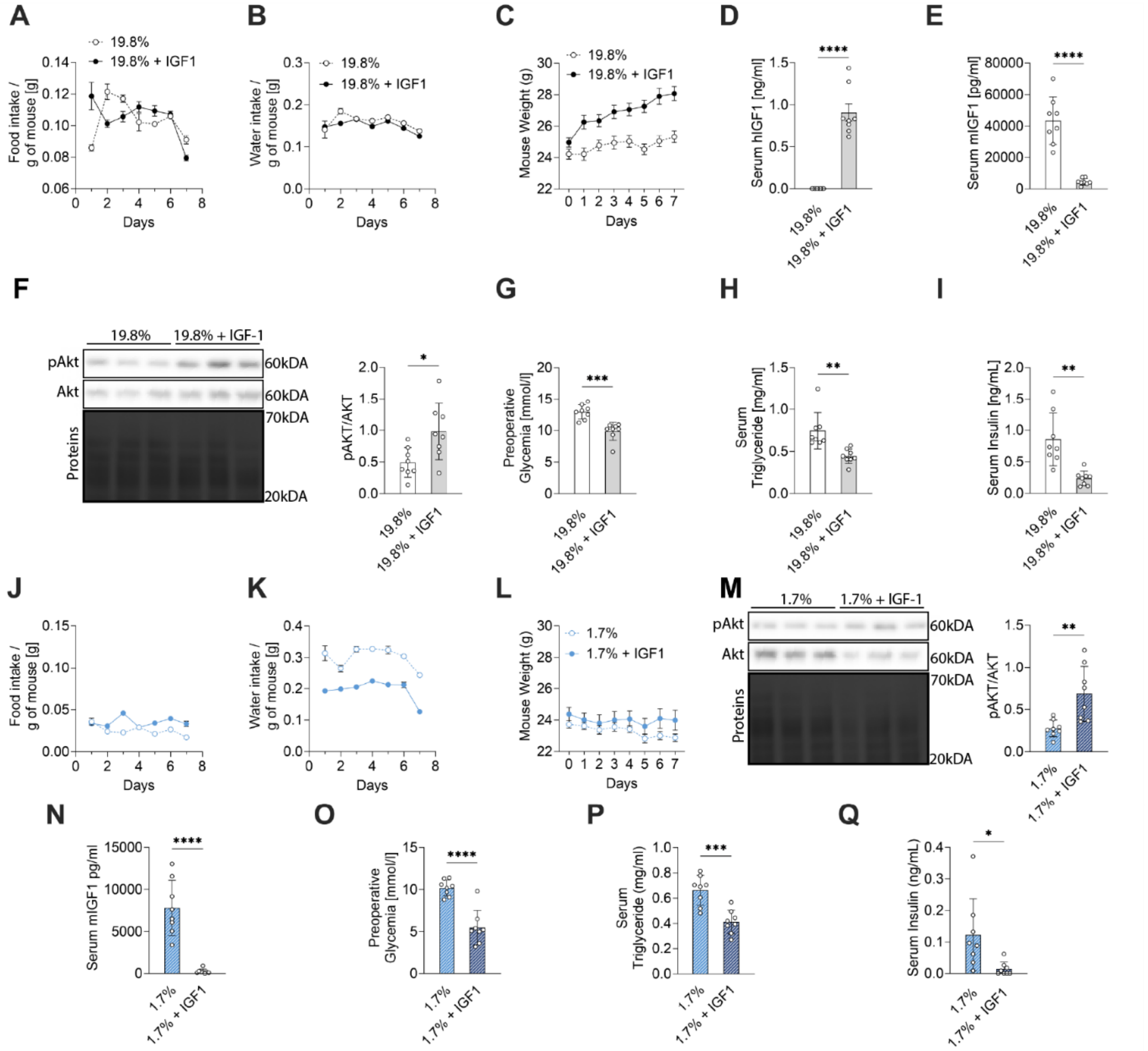

Figure S2. Additional data on severity of ischemia-reperfusion injury increased by IGF-1.

The following parameters were analyzed in 10-week-old male mice exposed to CTRL 19.8% protein diet and supplementation with vehicle or rhIGF-1 via osmotic pumps for one week:

(A) Food intake (normalized by body weight).

(B) Water intake (normalized by body weight).

(C) Mouse body weight.

(D) Serum human IGF-1 concentration.

(E) Serum mouse IGF-1 concentration.

(F) Western blot (left) and quantification (right) of AKT and pAKT in baseline kidneys (before IR injury)

(G) pre-operative glycemia.

(H) Serum triglycerides concentration.

(I) Serum insulin concentration.

The following parameters were analyzed in 10-week-old male mice exposed to LPHC 1.7% protein diet and supplementation with vehicle or rhIGF-1 via osmotic pumps for one week:

(J) Food intake (normalized by body weight).

(K) Water intake (normalized by body weight).

(L) Mouse body weight.

(M) Western blot (left) and quantification (right) of AKT and pAKT in baseline kidneys (before IR injury)

(N) Serum mouse IGF-1 concentration.

(O) pre-operative glycemia.

(P) Serum triglycerides concentration.

(Q) Serum insulin concentration.

*p values for D-I and M-Q were calculated with unpaired two-tailed T-test, *$p < 0.05$ **$p<0.01$ ***$p < 0.001$ ****$p < 0.0001$. *p* value for D < 0.0001, for E < 0.0001, for F = 0.0158, for G = 0.0003, for H = 0.0025, for I = 0.0012, for M = 0.0044, for N < 0.0001, for O < 0.0001, for P = 0.0003, and for Q = 0.0199. Sample sizes: n = 8 for all conditions. For A-C and J-L, data are shown as mean ± SEM; for D-I and M-Q, data are shown as mean ± SD. See also Main Data Figure 2.

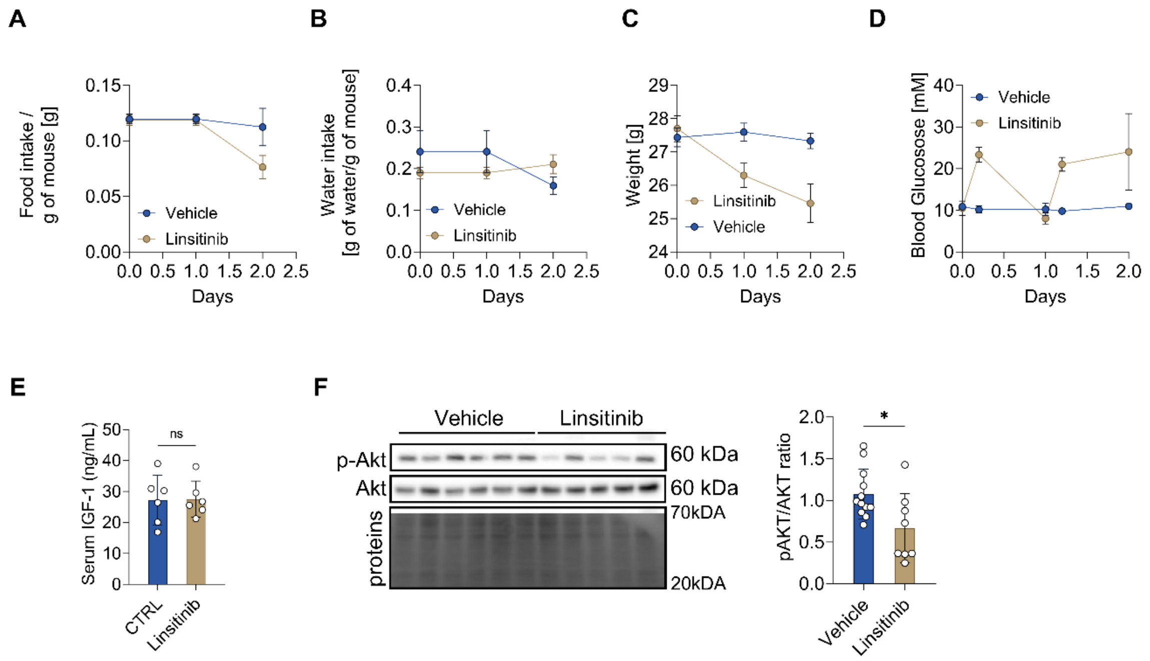

Figure S3. Additional data on reduction of ischemia-reperfusion injury by blockade of IGF-1 receptor.

(A) Food intake (normalized by body weight), (B) Water intake (normalized by body weight)

(C) Mouse body weight (D) glycemia at the indicated time in 10-week-old male mice treated vehicle or linsitinib.

(E) IGF-1 concentration in plasma from 10-weeks-old male mice after 2 days of treatment with vehicle or linsitinib.

(F) Western blot (left) and quantification (right) of AKT and pAKT in baseline kidneys (before IR injury) from 10-week-old male mice treated with vehicle or linsitinib.

*p values for E-F were calculated with unpaired two-tailed T-test, *$p < 0.05$ ***$p < 0.001$ ****$p < 0.0001$. *p* value for E = 0.9467, and for F = 0.0182. Sample sizes: n = 8 for all conditions. For A-D, data are shown as mean ± SEM; for E-F, data are shown as mean ± SD. See also Main Data Figure 3.

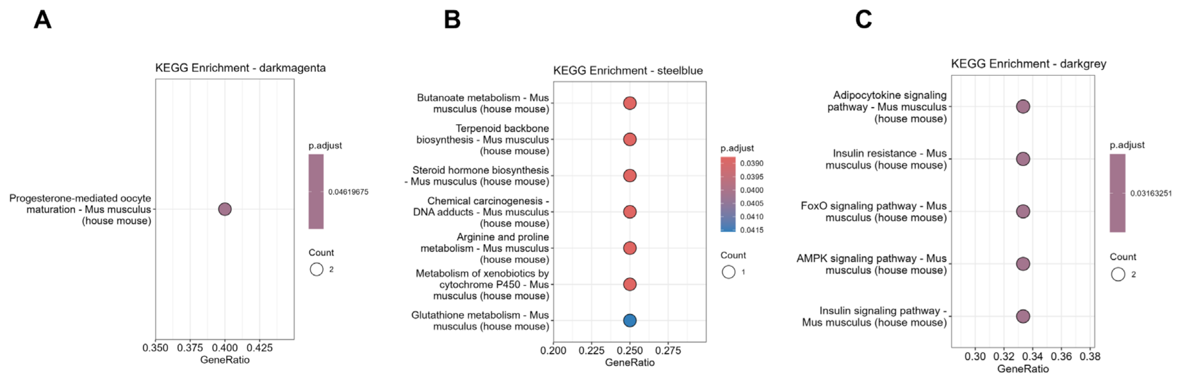

Figure S4. Additional data on bulk RNA sequencing analysis of gene and modules regulated by IGF1 that are associated with protection from ischemia-reperfusion.

Kyoto Encyclopedia of Genes and Genomes (KEGG) enrichment analysis of significantly positively correlated module (A) darkmagenta (B) steelblue (C) darkgrey with serum urea, creatinine, and hIGF-1.

Size of dots corresponds to the number of genes in the pathway, while color represents the significance level, with red indicating a low significance and blue a high significance. See also Main Data Figure 4.

SUPPLEMENTAL TABLES

**Experimental group**	**Mean protein intake [g/g of body weight]**	**Mean protein intake [% of total calory intake**	**Mean fat intake [g/g of body weight]**	**Mean fat intake [% of total calory intake**	**Mean carbohydrate intake [g/g of body weight]**	**Mean carbohydrate intake [% of total calory intake**
**Young Male CTRL**	0.089	19.8	0.047	10.4	0.31	69.9
**Young Male LP**	0.027	6.4	0.037	10.4	0.57	83.2
**Young Male HC**	0.038	3.3	0.042	1.4	0.46	95.3
**Young Male LPHC**	0.016	1.7	0.032	2.8	0.71	95.5
**Young Female CTRL**	0.089	19.8	0.047	10.4	0.31	69.9
**Young Female HC**	0.041	6.3	0.022	3.3	0.60	90.5
**Old Male CTRL**	0.049	19.8	0.18g	10.4	0.024	69.9
**Old Male HC**	0.023	5.2	0.007	2.7	0.38	92.1

Extended Data Table S1. Details on experimental diets.

Target	Specie	Forward Sequence 5'-3'	Reverse Sequence 5'-3'	product size
Krt20	mus musculus	ACAGTTCGAGAGACAGAGTCAA	CTGAGGTGGGACTGCAACTC	138
Rpl27a	mus musculus	TGATGTTGTTCGATCAGGCT	ATTTGGCCTTCACGATGACA	80
SPRR2F	mus musculus	ACTTTGGAGAACCTGATCCTGAG	GCACACCGAGGGAGAACAA	131

Extended Data Table S2. Primers for qPCR.
